# A pharmacist-led medication switch protocol in an academic HIV clinic: patient knowledge and satisfaction

**DOI:** 10.1186/s12879-018-3226-2

**Published:** 2018-07-06

**Authors:** Sarah S. Lee, Joshua P. Havens, Harlan R. Sayles, Jennifer L. O’Neill, Anthony T. Podany, Susan Swindells, Kimberly K. Scarsi, Sara H. Bares

**Affiliations:** 1 College of Medicine, University of Nebraska Medical Center, NE, Omaha, NE 68198-8106 USA; 2 Section of Infectious Diseases, University of Nebraska Medical Center, Omaha, NE USA; 3 College of Public Health, University of Nebraska Medical Center, Omaha, NE USA; 4 College of Pharmacy, University of Nebraska Medical Center, Omaha, NE USA

**Keywords:** HIV infection, Medication switch, Survey, Pharmacist, Patient knowledge and attitudes

## Abstract

**Background:**

Tenofovir alafenamide (TAF) is associated with less renal and bone toxicity compared with tenofovir disoproxil (TDF). TAF's recent FDA approval has spurred HIV providers to consider switching antiretroviral therapy (ART) regimens containing TDF to TAF to minimize long term risks. Patient views on the process of such medication switches have not been explored.

**Methods:**

Patients taking elvitegravir/cobicistat/emtricitabine/tenofovir disoproxil fumarate (E/C/F/TDF) following the Food and Drug Administration’s (FDA) approval of elvitegravir/cobicistat/emtricitabine/tenofovir alafenamide (E/C/F/TAF) received medication education from an HIV pharmacist prior to switching to the tenofovir alafenamide (TAF) formulation. Patients were asked to complete a cross-sectional survey assessing satisfaction with the switch process and knowledge about the new medication 4 to 8 weeks post-switch.

**Results:**

Sixty five patients completed the switch and 57 (88%) completed a follow-up survey. Most (86%) reported understanding why the switch was made, while 91% correctly identified that TAF is associated with reduced renal toxicity, and 73% correctly identified that TAF is associated with reduced bone toxicity. No statistically significant difference was found in satisfaction with or understanding of why the medication switch was made when assessed by sex, age, race, or education, but there was a trend toward significance in the distribution of answers based on education level with those with a high school diploma, General Educational Development (GED) or less being more likely to be satisfied with the medication switch (*p* = 0.074).

**Conclusions:**

Education from an ambulatory clinic-based HIV pharmacist resulted in high rates of patient satisfaction and understanding of the switch from TDF to TAF-containing ART.

**Electronic supplementary material:**

The online version of this article (10.1186/s12879-018-3226-2) contains supplementary material, which is available to authorized users.

## Background

Currently available antiretroviral therapy (ART) regimens have led to marked declines in HIV-related morbidity and mortality [1–3]. As a result, clinical attention is increasingly focused on choosing ART regimens that optimize tolerability, long-term safety, and efficacy.

Elvitegravir/cobicistat/emtricitabine/tenofovir disoproxil fumarate (E/C/F/TDF) is a widely-used fixed dose combination tablet recommended as first-line therapy for treatment-naïve HIV-1 infected adults and adolescents [1]. Although E/C/F/TDF has a favorable safety and tolerability profile, the TDF component carries risk of nephrotoxicity and has been shown to result in a greater decline in bone mineral density (BMD) than abacavir, the other recommend nucleoside transcriptase inhibitor [1, 4, 5].

In November 2015, the U.S. Food and Drug Administration (FDA) approved elvitegravir/cobicistat/emtricitabine/tenofovir alafenamide (E/C/F/TAF) for both HIV treatment naïve and experienced patients. TAF is a pro-drug of tenofovir that achieves higher intracellular concentrations of its active metabolite, tenofovir diphosphate, and results in lower plasma tenofovir concentrations compared with tenofovir disoproxil fumarate (TDF). Switching from TDF to TAF is associated with an improvement in proteinuria and renal biomarkers and smaller declines in bone mineral density [5]. This is particularly advantageous in people with underlying bone and kidney disease or those at high risk for these conditions. Importantly, these benefits have been seen without any compromise in virologic efficacy [1, 6–8].

While some benefits for switching from TDF to TAF-based ART regimens have been demonstrated, the ideal method for making the switch is less clear. HIV specialized pharmacists are increasingly involved in patient care, providing a significant amount of the medication teaching in HIV clinics across the United States and monitoring adherence with interventions when indicated [9]. Community pharmacy and clinic pharmacist-led HIV medication therapy management programs have resulted in improvements in medication adherence and reductions in medication errors [10–12]. Having pharmacists initiate predetermined medication switches provides efficiency for the health care team. The majority of respondents in a study exploring patient views of a pharmacist-led statin switch process were satisfied with the switch and indicated a preference for face-to-face consultation regarding the switch [13]. We are not aware of any data regarding preferred switch methods in the HIV literature.

In anticipation of switching patients from E/C/F/TDF to E/C/F/TAF, we designed a pharmacist-led switch protocol and survey to assess patient knowledge and opinions regarding the switch. We hypothesized that the pharmacist-led switch protocol would result in high levels of patient knowledge and satisfaction.

## Methods

The purpose of this research was to implement the switch from E/C/F/TDF to E/C/F/TAF using clinic-based pharmacists. The primary objective of the study was to assess patient satisfaction with the method in which the medication switch from E/C/F/TDF to E/C/F/TAF was made. Patient knowledge of the reason(s) why the medication switch was made was also assessed.

### Participant characteristics

Inclusion criteria for the study were that potential participants were attending the University of Nebraska Medical Center (UNMC) HIV clinic, were adults who speak English, were currently receiving E/C/F/TDF and were willing and able to provide informed consent. Those with HIV RNA level more than 50 copies/mL after at least 6 months on antiretroviral therapy, or receiving concomitant therapy contraindicated with E/C/F/TAF were excluded [14].

### Study procedures

Potentially eligible patients were either approached by the clinical pharmacist at the time of their clinic appointment or contacted by telephone to discuss the switch from E/C/F/TDF to E/C/F/TAF. Patients who were contacted by phone were given the option of either coming to the clinic for a face-to-face meeting with the clinical pharmacist or receiving information regarding the medication switch over the phone. As part of the pharmacist-led switch protocol, the clinical pharmacist assessed drug-drug interactions, provided medication and adherence counseling, prescribed the new medication under a collaborative drug therapy management (CDTM) agreement, and updated the medical record. Visits with the clinical pharmacist took place independently of visits with physicians and advanced practice providers. A brief written handout containing information about E/C/F/TAF and outlining the benefits it provides over E/C/F/TDF was offered to each participant. Following the medication switch, participants were invited to complete a study survey (see Additional file 1). The survey, designed to assess their knowledge and satisfaction with the switch process, was completed either in person or by telephone 4 to 8 weeks after the medication switch was made.

### Survey instrument

An 18-item survey consisting of closed-ended questions was designed by the investigators and modeled after a similar study of patient views regarding implementation of a statin medication switch [13]. The purpose of the survey was to assess patient knowledge and opinions regarding the medication switch. Demographic domains included gender, age, education level, insurance, and payer for antiretroviral therapy. Satisfaction and knowledge questions regarding the medication switch were assessed using a five-point Likert scale of agreement/disagreement.

### Statistical analysis

Basic descriptive statistics (frequencies and percentages) were calculated for all survey questions. Demographic characteristics of respondents were examined for the entire sample while the primary outcomes of interest, including satisfaction with the method in which the medication switch was made and knowledge regarding why the switch was made, were examined both overall and by demographic subgroups within the sample. Exact Pearson chi-square tests were used to assess the associations between satisfaction, knowledge, and all demographic variables except for education level, for which an exact Mantel-Haenszel chi-square test was used. *P*-values less than 0.05 were considered significant.

## Results

### Participant demographic data

A total of 65 patients underwent the medication switch from E/C/F/TDF to E/C/F/TAF, and 57 (88%) completed the follow-up survey between March 16, 2016 and August 16, 2016. The demographic characteristics of the respondents are outlined in Table 1. The majority of respondents were male (89%), white (60%), and at least 40 years of age (53%). Most (82%) reported some college education or higher. Greater than half (63%) were privately insured, while 24% were uninsured and received medications from the Nebraska AIDS Drug Assistance Program. Most (44, 77%) participants reported being informed of the switch in person, 5 (9%) of the participants reported being informed of the switch over the telephone, and 7 (12%) reported being informed of the switch both by telephone and in person.

**Table 1:** Characteristics of survey respondents (*n* = 57)

	n (%)
Sex	
Male	51 (89)
Female	6 (11)
Age Group	
< 40 years	27 (47)
≥ 40 years	30 (53)
Race	
Non-Hispanic White	35 (61)
Non-Hispanic Black	13 (23)
^a^Other	9 (16)
Education	
HS diploma or GED or below	11 (19)
Some college or 2 year associate’s degree	33 (58)
Bachelor’s degree or greater	13 (23)
Health Insurance	
Private	23 (40)
Uninsured	14 (24)
ADAP-sponsored health insurance	13 (23)
Medicaid/Medicare	5 (9)
Unknown	2 (4)

^a^Other includes Asian/Pacific Islander, White and Native American, White and Hispanic, and Black and Native American, as well as those reporting “Other”

*HS* high school, *GED* general education diploma

### Satisfaction and knowledge regarding switch

Overall, 54 (96%) of the participants agreed or strongly agreed that they were satisfied with how the switch was explained to them. Most (86%) reported understanding why the switch was made, 91% correctly identified that TAF is associated with reduced renal adverse effects, and 73% correctly identified that TAF is associated with reduced bone adverse effects, as outlined in Table 2. In regard to the brief handout that was given to all patients, only 40 (70%) respondents reported receiving written information about the new medication. Of those, 33 (83%) said that the written information was helpful.

**Table 2:** Satisfaction and knowledge following the medication switch

	Strongly Agree or Agree n (%)
I am happy that my Stribild was switched to Genvoya.	40 (70)
I am happy with the way in which the switch was explained to me.	54 (96)
I do not understand why my Stribild has been switched.	8 (14)
My medication was switched because Genvoya is better for my kidneys than Stribild.	51 (91)
My medication was switched because Genvoya is better for my bones than Stribild.	41 (73)
Genvoya and Stribild are both equally effective in treating HIV.	47 (82)

### Relationship between demographic variables and knowledge and satisfaction

There was no statistically significant difference in satisfaction with or understanding of why the medication switch was made when assessed by sex, age, race, or education, but there was a trend toward significance in the distribution of answers based on education level, with those with a high school diploma, General Educational Development (GED) or less being more likely to be satisfied with the medication switch (*p* = 0.074). Respondents receiving Medicaid or Medicare were statistically less likely to understand why their E/C/F/TDF had been switched (*p* = 0.037) compared to survey respondents with other forms of health insurance.

## Discussion

Our study evaluated patient satisfaction and knowledge following a pharmacist-led medication switch protocol. Overall, patient satisfaction with the switch process was high. Similarly, patient-reported and objective knowledge levels about the reason for the switch were high. This demonstrates the successful introduction of a pharmacist-led medication switch strategy in an HIV clinic. We aim to use the results to inform the implementation of future medication switch protocols.

While age, gender, race and education were not independently associated with knowledge and/or satisfaction with the medication switch, there was a trend toward significance in the distribution of answers based on education level, with those having less education being more likely to be satisfied with the medication switch. Although those receiving Medicaid/Medicare were less likely to understand the reason for their medication switch, the small number (*n* = 5) reporting this form of insurance limits the interpretation of these results.

Patients were given the option of receiving medication teaching by telephone, but most (89%) opted to receive the information in person, or over the telephone in addition to an in-person consultation. This is consistent with the results of other medication switch studies [13].

There were several limitations to this study. The survey was conducted at a single site and included a small number of participants as well as few women and young (age < 27 years old) participants, which limits the overall generalizability of the results. Additionally, while it would have been preferable for all respondents to complete the survey either in person or by phone, we offered both options in order to increase the response rate. This may have resulted in variability in the understanding or interpretation of survey questions as well as reporting of results based on the method of survey completion.

## Conclusions

We conclude that patient education from an ambulatory clinic-based HIV specialist pharmacist resulted in high rates of patient satisfaction and understanding of the switch from TDF to TAF-containing ART. The high rates of satisfaction and knowledge in this pharmacist-led medication switch protocol suggest that pharmacists could lead similar medication switches in the future yielding similar results, and face-to-face consultation should be considered given patient preference for this mode.

## Additional file

Additional file 1: Patient Survey. 18-item survey tool designed to assess patient knowledge and satisfaction regarding the switch from E/C/F/TDF to E/C/F/TAF. (PDF 23 kb)

## Abbreviations

ART: Antiretroviral therapy
CDTM: Collaborative drug therapy management
E/C/F/TAF: Elvitegravir/cobicistat/emtricitabine/tenofovir alafenamide
E/C/F/TDF: Elvitegravir/cobicistat/emtricitabine/tenofovir disoproxil fumarate
FDA: Food and Drug Administration
GED: General Educational Development
TAF: Tenofovir alafenamide
TDF: Tenofovir disoproxil fumarate
UNMC: University of Nebraska Medical Center
