# Supplementary material for: A pharmacist-led medication switch protocol in an academic HIV clinic: patient knowledge and satisfaction
Source: BMC Infect Dis. 2018 Jul 6;18:310. doi: 10.1186/s12879-018-3226-2 (PMC6035459; doi:10.1186/s12879-018-3226-2)
Supplement: Supplementary file 1 — Patient Survey. 18-item survey tool designed to assess patient knowledge and satisfaction regarding the switch from E/C/F/TDF to E/C/F/TAF. (PDF 23 kb) [file 12879_2018_3226_MOESM1_ESM.pdf]

PID: | | | | | | | |

STRIBILD SWITCH - Survey

- Complete this form using pen or pencil (not felt-tip marker).
- Print only one character/number per box.

Correct: ⊗ 9 8 Incorrect: 9 8

Please complete all sections of this questionnaire which is about the recent switch to your HIV medication

Section 1: How you learned about the change from Stribild to Genvoya

1. Did anyone talk (in person or on the phone) with you about switching your HIV medications prior to the switch?

- ◯ Yes ***--> Continue to question 2***
- ◯ No ***--> Skip to question 4***
- ◯ I don't know/I can't remember ***--> Skip to question 4***

2. How were you informed of the medication switch? ***(Please check all that apply)***

- ☐ Phone call from a pharmacist, doctor or nurse
- ☐ In person conversation with a pharmacist, doctor or nurse
- ☐ Can't remember
- ☐ Other, please give details: ____________________

3. Who informed you of the medication switch? ***(Please check all that apply)***

- ☐ A pharmacist from the UNMC HIV clinic/Specialty Care Center
- ☐ A pharmacist NOT at the UNMC HIV clinic/Specialty Care Center (e.g. Walgreen's, Elmwood, CVS)
- ☐ Physician
- ☐ Nurse Practitioner
- ☐ Other, please give details: ____________________

4. Did you receive written information about the new medication?

- ◯ Yes ***--> Continue to question 5***
- ◯ No ***--> Skip to Section 2 (question 6)***
- ◯ I don't know/I can't remember ***--> Skip to Section 2 (question 6)***

5. Was the written information about the new medication helpful?

- ◯ Yes
- ◯ No
- ◯ I don't know/I can't remember

Please continue on to the next page...

Section 2: Your views about the change to your Stribild prescription

Please indicate how strongly you agree or disagree with each of the following statements.

	Strongly Agree	Agree	Neither Agree nor Disagree	Disagree	Strongly Disagree
6. I am **happy** that my Stribild was switched to Genvoya.	○	○	○	○	○
7. I am **happy** with the way in which the switch was explained to me.	○	○	○	○	○
8. I **do not** understand why my Stribild has been switched.	○	○	○	○	○
9. My medication was switched because Genvoya is **better for my kidneys** than Stribild.	○	○	○	○	○
10. My medication was switched because Genvoya **is better for my bones** than Stribild.	○	○	○	○	○
11. Genvoya and Stribild are both **equally effective** in treating HIV.	○	○	○	○	○

Section 3: About you

12. What is your gender?

○ Male
○ Female

13. What is your age?

○ 19-26
○ 27-39
○ 40-59
○ 60-64
○ 65 or older

14. What is your race? ***(Please check all that apply)***

☐ White or European American
☐ African American or Black
☐ Asian or Pacific Islander
☐ Native American
☐ Other, please specify: ____________________

Please continue on to the next page...

15. Are you Hispanic/Latino/Latina?

◯ Yes
◯ No

16. What is the highest level of education you have completed?

◯ Grades 1-8 or no formal schooling
◯ Grades 9-12
◯ High school diploma or GED
◯ Some college, no degree
◯ Two year associate's degree
◯ Bachelor degree from 4 year university
◯ Postgraduate or professional degree

17. Which of the following describes your current health insurance coverage? *(Please check all that apply)*

☐ Private health insurance
☐ Medicaid
☐ Medicare
☐ Ryan White Program
☐ ADAP
☐ I do not have health insurance
☐ I don't know

18. Who pays for your HIV medications? *(Please check all that apply)*

☐ I pay
☐ Private health insurance
☐ Medicare/Medicaid
☐ ADAP or Ryan White Program
☐ Pharmaceutical company (e.g. pharmacy assistance program)
☐ I don't take HIV medications
☐ I don't know

Thank you for your time, we appreciate it very much!
